# Supplementary material for: Computational Investigation of Chirality-Based Separation of Carbon Nanotubes Using Tripeptide Library
Source: Biomolecules. 2023 Jan 13;13(1):175. doi: 10.3390/biom13010175 (PMC9855824; doi:10.3390/biom13010175)
Supplement: Supplementary file 1 [file biomolecules-13-00175-s001.zip › biomolecules-2102951-supplementary.pdf]

Article

Computational Investigation of Chirality-Based Separation of Carbon Nanotubes Using Tripeptide Library

Shrishti Singh [1], Heena R. Divecha [2], Abimbola Ayoola [2], Marvin Xavierselvan [2], Jack Devlin [3] and Isaac Macwan [3,*]

[1] Department of Bioengineering, George Mason University, Fairfax, VA 22030, USA
[2] Department of Biomedical Engineering, University of Bridgeport, Bridgeport, CT 06604, USA
[3] Department of Electrical and Biomedical Engineering, Fairfield University, Fairfield, CT 06834, USA
* Correspondence: imacwan@fairfield.edu

Supplementary Figures

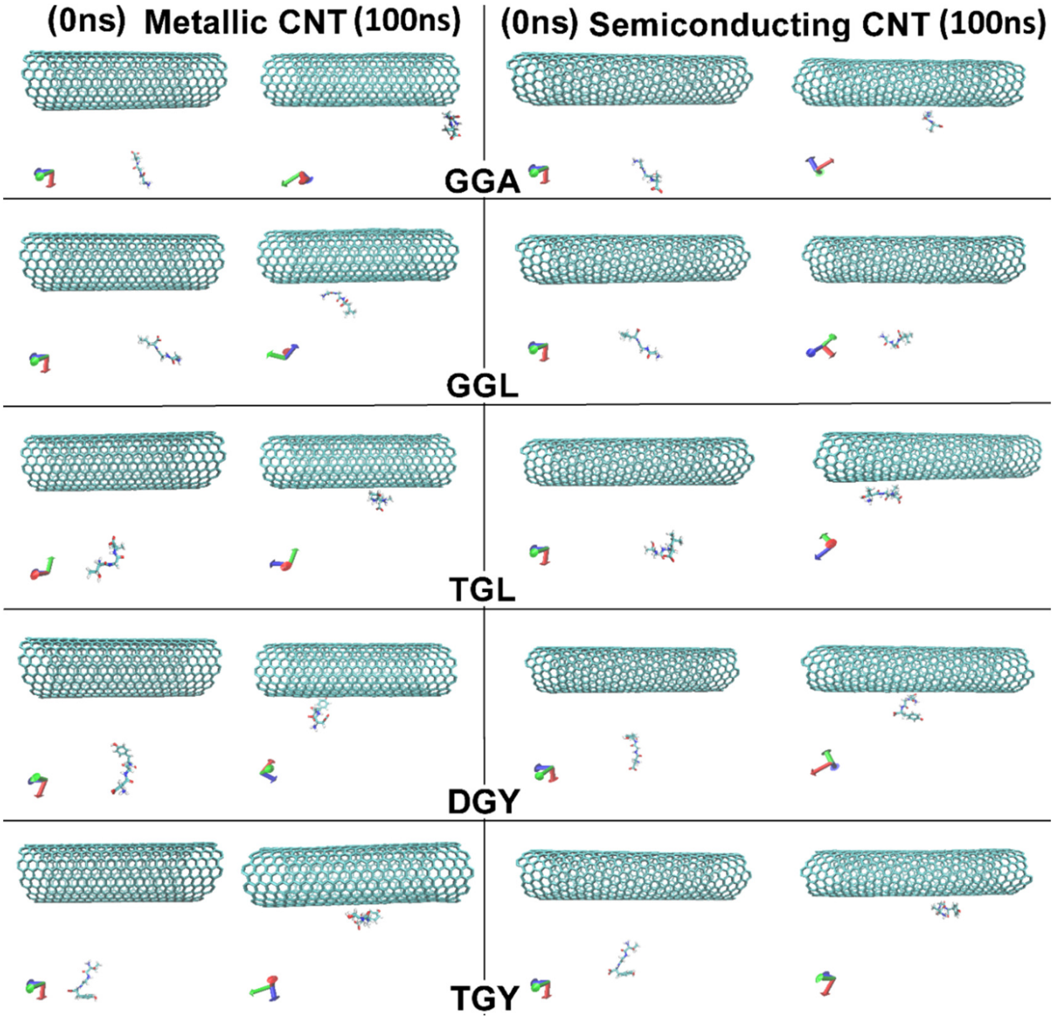

Figure S1. Trajectory screenshots showing the interactions of the five Group I tripeptides having at least one hydrophobic sidechain containing amino acid. Each block shows the first and the last frames for the simulation period of 100 ns for metallic (12, 12) and semiconducting (5, 15) CNTs.

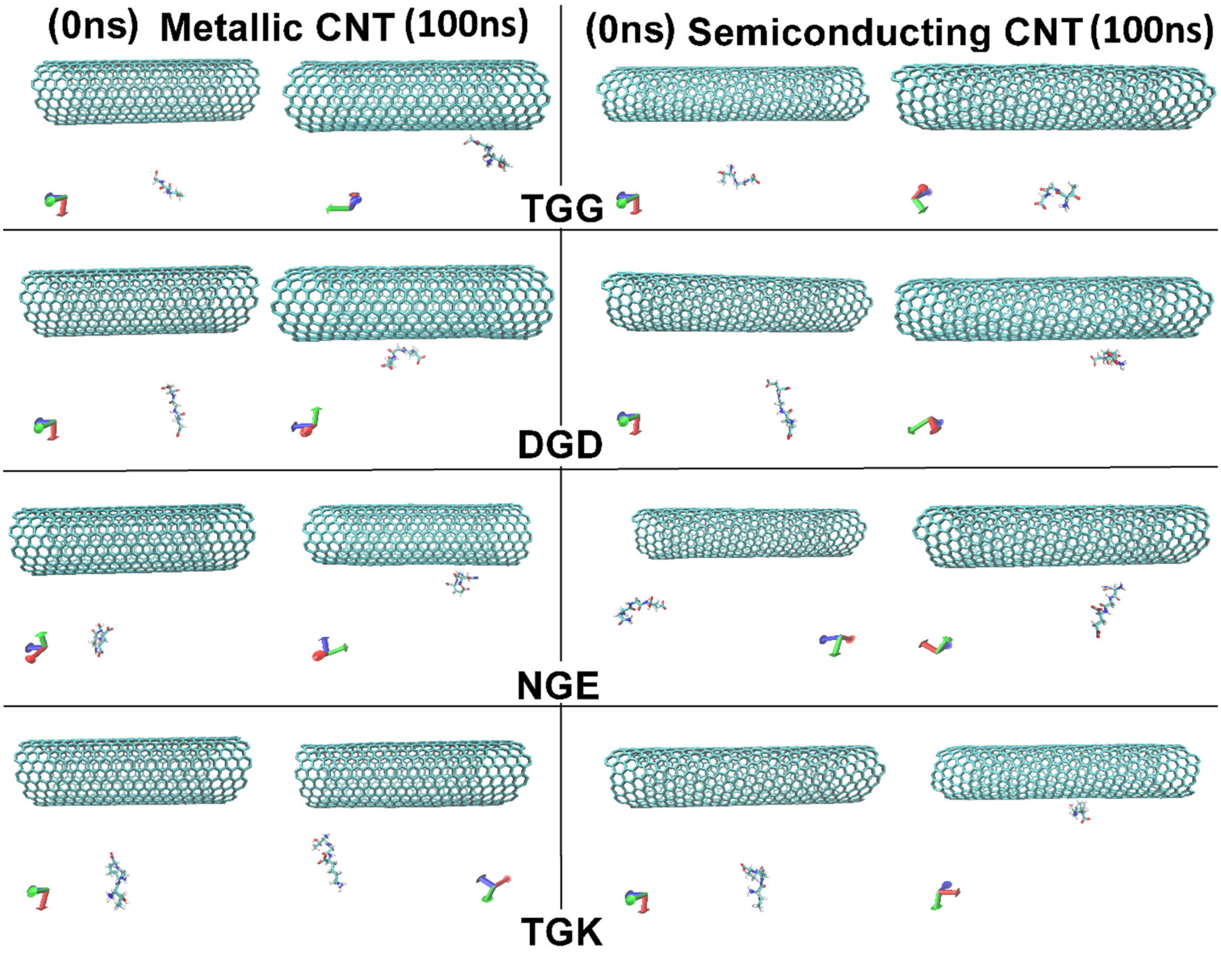

Figure S2. Trajectory screenshots showing the interactions of the four Group II tripeptides having no hydrophobic sidechain containing amino acids. Each block shows the first and the last frames for the simulation period of 100 ns for metallic (12, 12) and semiconducting (5, 15) CNTs.

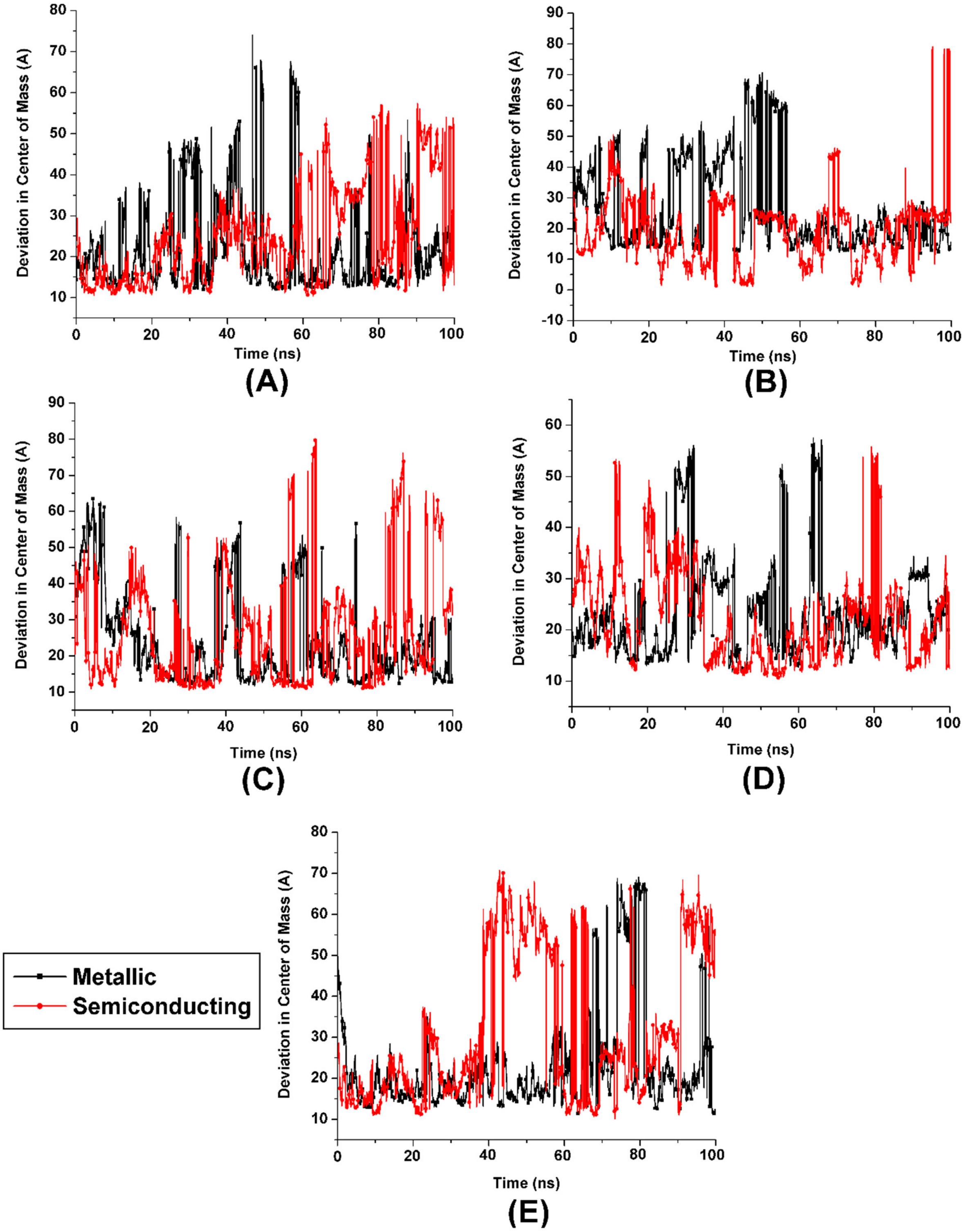

Figure S3. Center of Mass deviations for individual tripeptides of Group I with respect to the metallic and semiconducting CNTs: (**A**) GGA; (**B**) GGL; (**C**) TGL; (**D**) DGY and (**E**) TGY.

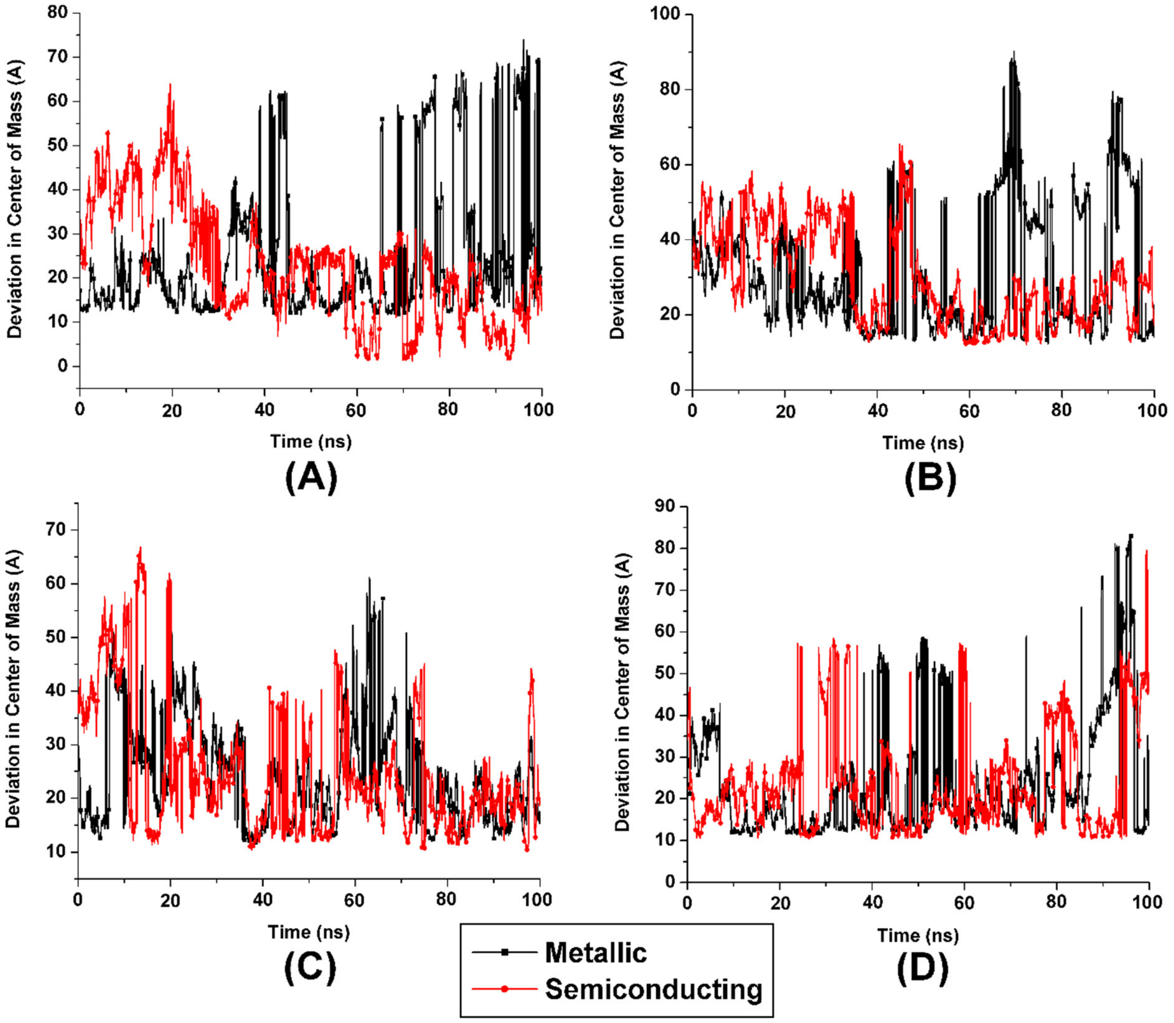

Figure S4. Center of Mass deviations for individual tripeptides of Group II with respect to the metallic and semiconducting CNTs: (**A**) TGG; (**B**) DGD; (**C**) NGE and (**D**) TGK.

Supplementary Tables

Table S1. Physical properties of tripeptides used in this study and their codes based on the international nucleotide sequence database.

Tripeptide codes	Tripeptides	Nature
DGD	Aspartic acid-Glycine-Aspartic acid	Acidic- Neutral-Acidic<br>Polar-Polar-Polar
DGY	Aspartic acid-Glycine-Tyrosine	Acidic-Neutral-Neutral<br>Polar-Polar-Polar (aromatic)
GGA	Glycine-Glycine-Alanine	Neutral-Neutral-Neutral<br>Polar-Polar-Nonpolar (hydrophobic)
GGL	Glycine-Glycine-Leucine	Neutral-Neutral-Neutral<br>Polar-Polar-Nonpolar
NGE	Asparagine-Glycine-Glutamic acid	Neutral-Neutral-Acidic<br>Polar-Polar-Polar
TGG	Threonine-Glycine-Glycine	Neutral-Neutral-Neutral<br>Polar-Polar-Polar
TGK	Threonine-Glycine-Lysine	Neutral-Neutral-Basic<br>Polar-Polar-Polar
TGL	Threonine-Glycine-Leucine	Neutral-Neutral-Neutral<br>Polar-Polar-Nonpolar
TGY	Threonine-Glycine-Tyrosine	Neutral-Neutral-Neutral<br>Polar-Polar-Polar (aromatic)

Table S2. Physical properties of each amino acid, which form the flanking residue of glycine in the tripeptide library. Glycine observed to be neither polar nor hydrophobic with its functional sidechain being a hydrogen atom exhibits achiral symmetry.

**Amino acids**	**Solubility and charge**
Threonine (T)	Polar, uncharged sidechain
Tyrosine (Y)	Hydrophobic sidechain
Glycine (G)	Special case (can be polar or hydrophobic)
Aspartic acid (D)	Polar, negatively charged sidechain, acidic
Glutamic acid (E)	Polar, negatively charged sidechain, acidic
Leucine (L)	Hydrophobic sidechain
Lysine (K)	Polar, positively charged sidechain, basic
Alanine (A)	Hydrophobic sidechain
Asparagine (N)	Polar, uncharged sidechain
